# Retraction: Arctigenin inhibits the progression of colorectal cancer through epithelial-mesenchymal transition via PI3K/Akt/mTOR signaling pathway

**DOI:** 10.1371/journal.pone.0341384

**Published:** 2026-01-26

**Authors:** The PLOS One Editors

Following the publication of this article [1], concerns were raised regarding Figs 3, 4, and 6–8. Specifically:

- In Figs 3 and 4, there appears to be partial overlap between:
  - The panels “Control” (after rotation), “Arctigenin 1 µM”, and “Arctigenin 10 µM” in Fig 3A and the respective panels in Fig 4A,
  - The panels “Control”, “Arctigenin 1 µM”, “Arctigenin 5 µM” and “Arctigenin 10 µM” in Fig 3B and the respective panels in Fig 4B.
  - The panels “Arctigenin 1 µM” and “Arctigenin 10 µM” in Fig 3C and the respective panels in Fig 4C.
- In Fig 6C, the “Arctigenin, 10 µM” panels appear similar to the “12µM” panels in Fig 5B in [2,3], and the “Control” panels in Fig 6B in [2,3].
- In Fig 8, there appears to be overlap between:
  - The “control” panels in Figs 8E and 8F.
  - The panels “Arctigenin 5 µM” and “Arctigenin 5 µM + 740YP 10 µM” in Fig 8E and the respective panels in Fig 8F.

Furthermore, PLOS noted that details on the solvent used for arctigenin and critical controls were not included for the reported *in vitro* and *in vivo* experiments where the effect of arctigenin was evaluated.

The corresponding author, PF, stated that errors were made when the image data were uploaded.

In light of the extent of the above unresolved concerns that question the reliability and integrity of the results presented in [1], the *PLOS One* Editors retract this article.

Since the publication of [1], Reference 5 [4] has been retracted [5].

PF responded but expressed neither agreement nor disagreement with the editorial decision. XFC, PGL, NS, XSL, RKH, and LXZ either did not respond directly or could not be reached.
